# A cardiovascular disease risk factor in children with congenital heart disease: unmasking elevated waist circumference - a CHAMPS* study *CHAMPS: Children’s Healthy-Heart Activity Monitoring Program in Saskatchewan

**DOI:** 10.1186/s12872-020-01508-y

**Published:** 2020-05-19

**Authors:** Erin Barbour-Tuck, Natasha G. Boyes, Corey R. Tomczak, Dana S. Lahti, Chantelle L. Baril, Charissa Pockett, Shonah Runalls, Ashok Kakadekar, Scott Pharis, Timothy J. Bradley, Kristi D. Wright, Marta C. Erlandson

**Affiliations:** 1 Department of Pediatrics, College of Medicine, University of Saskatchewan, Saskatoon, Canada; 2 College of Kinesiology, University of Saskatchewan, 87 Campus Drive, Saskatoon, Saskatchewan S7N 5B2 Canada; 3 Department of Psychology, University of Regina, Regina, Canada

**Keywords:** Congenital heart disease, Abdominal obesity, Physical activity, Cardiometabolic risk, Waist circumference

## Abstract

**Background:**

Children with congenital heart disease (CHD) have an elevated risk of future cardiovascular disease but the underlying mechanisms are unclear. Abdominal obesity (measured as waist circumference) is a risk factor for adult onset of cardiovascular diseases and is correlated with low physical activity levels, commonly found in children with congenital heart disease. Elevated waist circumference may be a mechanism by which cardiovascular disease risk is elevated in children with CHD. The purpose of this study was to compare waist circumference between children with and without CHD, while considering potential confounders. We hypothesized that children with CHD would have higher measures of waist circumference when controlling for differences in birthweight, lean mass, and physical activity.

**Methods:**

Thirty-two children with CHD (10.9 ± 2.6 years; 12 female) from the Children’s Healthy-Heart Activity Monitoring Program in Saskatchewan, and 23 healthy controls (11.7 ± 2.5 years; 10 female) were studied. Waist circumference, physical activity (physical activity questionnaire), body composition (lean mass; dual x-ray absorptiometry), and birthweight were assessed. Analysis of covariance, Mann-Whitney U, and independent sample *t*-tests were used to assess group differences (*p* < 0.05).

**Results:**

Children with CHD had greater waist circumference than controls, controlling for lean mass, physical activity, birthweight, and sex (*F* (1, 49) = 4.488, *p* = 0.039). Physical activity, lean mass, and birthweight were not significantly different between groups (*p* > 0.05).

**Conclusion:**

Our findings generate a novel hypothesis—higher waist circumferences in children with CHD compared to age-matched controls, may contribute to an elevated risk of cardiovascular disease.

## Background

Adults with congenital heart disease (CHD) may have a greater risk than their healthy peers for developing cardiovascular and metabolic diseases such as type II diabetes and atherosclerosis [1–4]. Preclinical markers and risk factors of these diseases (e.g.*,* hypertension, dyslipidemia, dysglycemia, arterial stiffness, and low exercise capacity) arise during childhood in association with elevated adiposity and waist circumference, and low physical activity [5–10]. The prevalence of low physical activity, overweight, and obesity in children with CHD [11–15] may account for disparities in preclinical markers in childhood and cardiovascular and metabolic diseases in this population later in life [16]. The relationships between physical activity, body fat, and body mass index (BMI) with health are ubiquitous; however, they do not entirely explain the observed differences in preclinical markers or fitness between children with CHD and healthy peers. Identifying other contributing factors to cardiovascular and metabolic risk is important for preventing adult disease in individuals with CHD.

Elevated waist circumference, a surrogate measure of abdominal fat deposition, may be a contributing factor in the process linking CHD to adult cardiovascular disease. Abdominal fat is highly metabolically active and produces elevated levels of circulating blood lipids [17] and inflammatory cytokines such as tumor necrosis factor-α and interleukins [18]. In healthy populations, waist circumference has been linked to low birthweight, catch-up growth, and low physical activity; further, there are longitudinal relationships between birthweight, early childhood growth, and childhood abdominal fat and adult cardiovascular health [7, 9, 10, 19–21]. Children with CHD are susceptible to low birthweight, catch-up growth, and low physical activity [22–24]. Therefore, elevated waist circumference during childhood may pose a similar cardiovascular disease risk to children with CHD as it does in other populations, but to date this has not been properly explored.

Studies in children with CHD often measure only body fat or identify overweight by BMI, and conclude that there are no differences in fatness between CHD and healthy children contributing to the differences in health markers (i.e.*,* arterial stiffness, fitness, etc.) [14, 16], but these observations may be incomplete. Waist circumference is often overlooked in the analyses [25], or the analyses do not account for key potential moderators of waist circumference such as birthweight, sex, body size, and physical activity [16, 26]. The inclusion of waist circumference and its moderators may be critical to understanding the mechanisms behind the high rates of cardiometabolic diseases in this population. Therefore, the purpose of this study was to examine waist circumference between children with and without CHD, while considering potential confounders. We hypothesized that children with CHD would have higher measures of waist circumference compared to healthy age-matched controls.

## Methods

Study participants included 32 children (12 female) with CHD with an age range of 7–16 years (10.9 ± 2.6 years) from the Children’s Healthy-Heart Activity Monitoring Program in Saskatchewan (CHAMPS), and 23 (10 females) age- and sex-matched healthy controls, with an age range of 7–16 years (11.7 ± 2.5 years). CHD conditions included Fontan circulation [*n* = 7; pre-Fontan circulation CHD included hypoplastic left heart syndrome (*n* = 4), hypoplastic right heart syndrome (*n* = 1), right ventricular single ventricle with transposition of the great arteries and pulmonary stenosis (*n* = 1), and tricuspid atresia with multiple ventricular septal defects (*n* = 1)], tetralogy of Fallot (*n* = 5), heart transplantation [*n* = 4; underlying heart lesion of dilated cardiomyopathy (*n* = 2), hypoplastic left heart syndrome (*n* = 1), and tricuspid atresia with pulmonary stenosis (*n* = 1)], transposition of the great arteries (*n* = 3), septal defects (*n* = 3; 1 atrial and 2 ventricular), coarctation of the aorta (*n* = 2), cardiomyopathy (*n* = 2), double outlet right ventricle (*n* = 2), pulmonary stenosis (*n* = 2), aortic stenosis (*n* = 1), and tricuspid atresia (*n* = 1). Of the children with CHD, 31 were post-operative (mean time post-operative 7.3 ± 4.1 years) and 1 had not undergone surgical repair or device implantation. CHD diagnoses and surgical details are further described in Table 1. Children in our sample were ethnically similar, with 87% of those with CHD and 100% of controls self-identifying as European-white. Four children (13%) identified as other than European-white: one as Caucasian/Filipino, one as Métis, one as Chinese, one as Caucasian/Latino (Spanish). The study population included all children from the larger CHAMPS cohort (*n* = 38) with complete waist circumference, physical activity and body composition data. Ethical approval was obtained from the University of Saskatchewan’s Biomedical Research Ethics Board and written parental consent and child assent were obtained prior to testing.

**Table 1:** Diagnostic and surgical features of children with CHD

CHD #	Age at Study (years)	Age at Surgery	Time since Surgery (years)	Weight at Study (kg)	Weight at Surgery (kg)	NYHA Class	Diagnosis	Surgical Procedure	Medications
1	9	11 days	9	31.5	Unknown	I	Pulmonary stenosis	Balloon valvuloplasty	–
2	10	N/A	N/A	34.5	N/A	I	Idiopathic dilated cardiomyopathy	None	ASA, Carvedilol, Enalapril, Spironolactone
3	14	^a^Neonate; ^b^9 years; ^c^13 years	1	48.0	Unknown	I	Aortic stenosis	^a^Balloon valvuloplasty; ^b-c^Aortic valve reconstruction × 2	ASA, Ranitidine, Warfarin
4	10	^a^1 day; ^b^10 days; ^c^2 years; ^d^4 years	6	33.0	^d^15.8	I	Transposition of the great arteries	^a^Balloon atrial septostomy; ^b^Arterial switch; ^c^Balloon branch pulmonary artery stenosis; ^d^Right ventricular outflow tract patch	–
5	12	3 months	12	48.0	4.5	I	Tetralogy of Fallot	Tetralogy of Fallot repair	Fluoxetine, Lisdexamfetamine, Melatonin
6	14	^a^8 days; ^b^10 months; ^c^3 years; ^d^6 years	8	38.7	^d^18.8	I	Hypoplastic left heart syndrome	^a^Norwood/BT shunt; ^b^Glenn; ^c^Fontan; ^d^Tricuspid valve reconstruction	ASA, Enalapril
7	9	6 months	8.5	28.0	7.6	I	Double outlet right ventricle, atrial septal defect	Atrial and ventricular septal defect patches, right ventricular muscle bundle resection, main pulmonary artery arterioplasty	–
8	9	6 months	8.5	35.0	7.2	I	Tetralogy of Fallot	Ventricular septal defect patch, right ventricle muscle bundle resection, pulmonary valvuloplasty	ASA
9	13	^a^2 weeks; ^b^6 months; ^c^2.75 years	10	57.0	Unknown	I	Hypoplastic left heart syndrome	^a^Norwood/BT shunt; ^b^Glenn; ^c^Fontan	ASA
10	8	18 months	6.5	27.0	12.0	I	Idiopathic dilated cardiomyopathy	Heart transplant	Atorvastatin, Cyclosporine, Enalapril, Mycophenolic acid, Vitamin D
11	14	^a^3 months; ^b^2 years	12	44.0	Unknown	I	Tricuspid atresia, ventricular septal defect	^a^Glenn; ^b^Fontan	ASA, Norethisterone
12	9	^a^1 month; ^b^5 month	8.5	27.0	Unknown	I	Coarctation of the aorta	^a^End-to-end coarctation repair; ^b^Balloon recoarctation	–
13	16	12 years	4	135.0	83.0	I	Dilated/restrictive cardiomyopathy	Transplant	Atorvastatin, Mycophenolic acid, Tacrolimus, Vitamin D
14	9	^a^7 months; ^b^5 years	4	47.5	18.6	I	Pulmonary stenosis, atrial septal defect, tricuspid regurgitation	^a^Balloon valvuloplasty; ^b^Atrial septal defect closure and tricuspid valve repair	Fluticasone propionate, Salbutamol, Omega 3
15	8	7 years	1	21.8	Unknown	I	Congenitally corrected transposition of the great arteries	Pacemaker	ASA, Methylphenidate
16	13	10 years	3	35.5	32.0	I	Perimembraneous ventricular septal defect	Perimembraneous ventricular septal defect suture closure, right ventricular muscle bundle resection	–
17	10	^a^10 days; ^b^1 year; ^c^1 year	9	27.0	Unknown	IV	Tricuspid atresia, pulmonary stenosis	^a^Right BT shunt; ^b^Left BT shunt; ^c^Glenn;	ASA, Enalapril, Omeprazole
18	11	^a^2 weeks; ^b^5 months	10.5	33.0	^a^2.6; ^b^5.6	I	Tetralogy of Fallot	^a^Right BT shunt; ^b^Tetralogy of Fallot repair	–
19	10	^a^8 days; ^b^6 months; ^c^2.5 years	7	29.0	^a^3.2; ^b^7.1; ^c^14 .0	I	Hypoplastic left heart syndrome	^a^Norwood/Sano shunt; ^b^Glenn; ^c^Fontan	ASA, Enalapril
20	14	^a^2 weeks; ^b^1 year	13	46.0	Unknown	I	Coarctation of the aorta, ventricular septal defect	^a^Subclavian flap coarctation repair; ^b^Ventricular septal defect patch	–
21	10	2 months	10	21.0	Unknown	I	Tetralogy of Fallot	Tetralogy of Fallot repair	ASA
22	13	5 months	13	50.0	Unknown	I	Tetralogy of Fallot	Tetralogy of Fallot repair and pacemaker	–
23	13	^a^3 days; ^b^5 months; ^c^2 years	11	58.5	^a^3.8	I	Right atrial isomerism, right ventricular single ventricle, transposition of the great arteries, pulmonary stenosis	^a^Left BT shunt; ^b^Glenn; ^c^Fontan	ASA, Penicillin, Sotalol
24	14	^a^3 months; ^b^1 year	13	52.0	Unknown	I	Left atrial isomerism, double outlet right ventricle, pulmonary stenosis, right-sided aortic arch	^a^Left BT shunt; ^b^Full repair	–
25	12	^a^2 weeks; ^b^9 months; ^c^2.75 years	9	46.5	Unknown	I	Double outlet right ventricle, mitral atresia, hypoplastic left ventricle, interrupted aortic arch	^a^Aortic arch repair and pulmonary artery band; ^b^Glenn; ^c^Fontan	ASA
26	14	12 years	2	81.0	64.0	I	Hypertrophic cardiomyopathy	Implantable Cardioverter Defibrillator	Atenolol, Escitalopram, Medroxyprogesterone acetate Topiramate
27	8	4 years	4	27.5	17.4	I	Sinus venosus atrial septal defect	Atrial septal defect repair with Warden procedure	ASA
28	7	5 years	2	28.0	19.0	I	Ventricular septal defect, heart block	Ventricular septal defect patch and pacemaker	–
29	13	3 months	13	48.5	Unknown	I	Transposition greater arteries, atrial and ventricular septal defects	Arterial switch, coarctation repair, atrial septal defect suture, ventricular septal defect patch	–
30	7	^a^6 days; ^b^6 months; ^c^2 years; ^d^4 years	3	30.0	^a^4.1; ^b^7.6; ^c^14.1; ^d^19.2	I	Dextrocardia, double inlet left ventricle, hypoplastic right ventricle, transposition of the great arteries, pulmonary atresia, ventricular septal defect	^a^Right BT shunt; ^b^Glenn; ^c^Fontan; ^d^Pacemaker	ASA, Amantadine, Risperidone, Sertraline
31	7	^a^2 months; ^b^9 months; ^c^11 months; ^d^2 years; ^e^4.5 years	2.5	28.0	^d^12.9; ^e^18.0	I	Tricuspid atresia, normally related great arteries, pulmonary stenosis	^a^Right BT shunt; ^b^Left BT shunt; ^c^Glenn; ^d^Fontan; ^e^Transplant	Calcium, Fluticasone propionate, Methylphenidate, Mycophenolic Acid, Sertraline, Tacrolimus, Vitamins B2 and D
32	8	^a^10 days; ^b^5 months; ^c^4.5 years; ^d^7 years	1	22.5	^c^13.1; ^d^19.1	I	Hypoplastic left heart syndrome	^a^Norwood/Sano shunt; ^b^Glenn; ^c^Fontan; ^d^Transplant	Mycophenolic Acid, Tacrolimus, Vitamin D

CHD patient diagnosis, surgical procedure details, and medications at time of study. Multiple surgeries are lettered (a, b, c, etc.) and reported in the order of earliest to latest. *ASA* Acetylsalicylic acid, *CHD* Congenital heart disease, *NYHA* New York Heart Association

### Anthropometrics

Waist circumference was measured as the midway point between the lowest rib and the iliac crest, generally corresponding to the smallest circumference, and recorded to the nearest 0.1 cm [27]. Height and sitting height were measured on a wall mounted stadiometer (Holtain Ltd., Crosswell, UK) and recorded to the nearest 0.1 cm. Weight was taken on a calibrated digital scale (Toledo Scale Company of Canada, Windsor, ON) and recorded to the nearest 0.1 kg. Birthweight was obtained by parental report.

### Physical activity

Physical activity was assessed using the self-report 7-day recall Physical Activity Questionnaires for Children (7–13 years) or Adolescents (14–18 years) [28–30]. The Physical Activity Questionnaires for Children and Adolescents have demonstrated internal consistency and validity via moderate relationships with other 7-day activity recalls, teacher evaluations, and other measures of physical activity, including accelerometry [28–30]. The Physical Activity Questionnaires for Children and Adolescents use a 5-point Likert scale to yield a composite physical activity score, with a higher number indicating a greater level of physical activity. Participants were asked to recall their activities before school, during recess (elementary school only), lunch, after school, and after supper, and to select their general patterns of activity (i.e.*,* sat and talked with friends versus ran around and played most of the time) for the last week. A researcher provided participants with verbal instruction on how to complete the questionnaire and remained available to answer questions. Participants, in accordance with the author-recommended questionnaire protocol, completed questionnaires with minimal input from parents.

### Dual-energy x-ray absorptiometry (DXA)

DXA was used to assess body composition. A total body scan was taken in array mode by a certified technologist (QDR Discovery Wi; Hologic, Inc., Bedford, MD, USA) and scans were analyzed using QDR software for Windows XP (QDR Discovery, Hologic, Inc.) to assess total lean mass (g) and total fat mass (g), which were then used to calculate total % lean mass and total % fat mass.

### Waist circumference covariates

Factors modulating waist circumference (sex, birthweight, physical activity, and total lean mass) were included as covariates in our statistical model based on previous studies and relationships identified in other populations [20, 31–34]*.* Sex was included in the model because the overall sample was unbalanced with more males than females, despite sex-matching between groups. Birthweight was included because of the known relationship between low birthweight and cardiovascular risk factors including abdominal fat in children [20, 35]. Physical activity was included because of known relationships to abdominal fat and cardiovascular risk factors in children [9]. Lean mass was included in the model to control for body size independent of fat mass and to eliminate the assumption of BMI regarding identical fractional body compositions (i.e.*,* at a given BMI, not all individuals will have the same amount of fat mass) [34]. While DXA measures offer a more valid measure of abdominal fat, the selection of waist circumference as our dependent variable was deliberate as it is a more feasible, accessible, and non-invasive technique allowing for widespread use in pediatric clinics at no cost. Age was not used as a covariate given the control group was age-matched.

### Statistical analysis

Dependent variables were assessed for normality using the Shapiro-Wilk test. Group differences in descriptive characteristics, including overweight and obesity prevalence (based on pediatric BMI cutoffs [36]), were assessed using independent sample *t*-tests for parametric variables and Mann-Whitney U for non-parametric variables. Data are reported as means ± standard deviation. The relationship between waist circumference and each covariate in both CHD and control groups were assessed using Pearson bivariate correlations. Physical activity score and waist circumference were divided into quartiles and the group relationships to quartiles were assessed using Pearson Chi-square analyses. Finally, the difference in waist circumference between the CHD and control groups was assessed using an analysis of covariance with sex, birthweight, physical activity, and lean mass as covariates. All analyses were assessed using SPSS version 24.0 (Chicago, IL, USA) and significance was accepted when *p* < 0.05.

## Results

Child/adolescent descriptive characteristics by group are listed in Table 2. CHD and control groups were similar in age, birthweight, height, weight, BMI, total fat mass, total lean mass, percent fat mass, percent lean mass, and physical activity score (*p* > 0.05). Unadjusted waist circumference as indicated by the Mann-Whitney U test was not significantly different between CHD (mean rank = 28.5, sum of ranks = 913.0) and control groups (mean rank = 27.3, sum of ranks = 627.0; *U* = 351.0, *p* = 0.772).

**Table 2:** Child/adolescent descriptive characteristics by study group

	Control	CHD	*p*-value
N	23	32	–
Sex (M, F)	13, 10	20, 12	0.655^a^
Age (years)	11.7 ± 2.5	10.9 ± 2.6	0.268^b^
Birthweight (kg)	3.67 ± 0.58	3.38 ± 0.59	0.071
Height (m)	1.49 ± 0.17	1.44 ± 0.17	0.250
Weight (kg)	42.3 ± 15.3	41.3 ± 21.6	0.473^b^
BMI (kg/m^2^)	18.3 ± 3.1	18.9 ± 4.7	0.720^b^
Total fat mass (kg)	8.7 ± 5.1	10.4 ± 9.4	0.746^b^
Total lean mass (kg)	31.5 ± 10.5	29.1 ± 11.9	0.298^b^
Percent fat mass (%)	19.9 ± 5.3	22.8 ± 7.6	0.131^b^
Percent lean mass (%)	75.4 ± 5.4	73.1 ± 7.4	0.204
Physical activity score (1–5 scale)	3.35 ± 0.69	3.05 ± 0.71	0.117
Overweight/Obese prevalence	8%	17%	0.374

Data presented as means ± standard deviation. *BMI* Body mass index, *CHD* Congenital heart disease. Control, age- and sex-matched control group. ^a^Chi-square analysis; ^b^Mann-Whitney U analysis. No significant differences between groups

There were significant relationships between waist circumference and physical activity score in CHD (*r* = − 0.356, *p* = 0.045) and controls (*r* = − 0.691, *p* < 0.001); waist circumference and lean mass in CHD (*r* = 0.853, *p* < 0.001) and controls (*r* = 0.847, *p* < 0.001); and waist circumference and birthweight in CHD (*r* = 0.355, *p* = 0.046) but not controls (*p* = 0.057). There was no significant relationship between waist circumference and sex in either group (both *p* > 0.05).

There was no significant association between physical activity quartile and CHD/control group (x^2^ (3) = 5.036, *p* = 0.169) or between waist circumference quartile and CHD/control group (x^2^ (3) = 0.990, *p* = 0.804); however, there was a 26 cm difference in waist circumference identified between the top (56.8 ± 3.1 cm) and bottom quartile (83.2 ± 13.9 cm) of physical activity when groups were combined (*p* < 0.001).

**Fig. 1:**
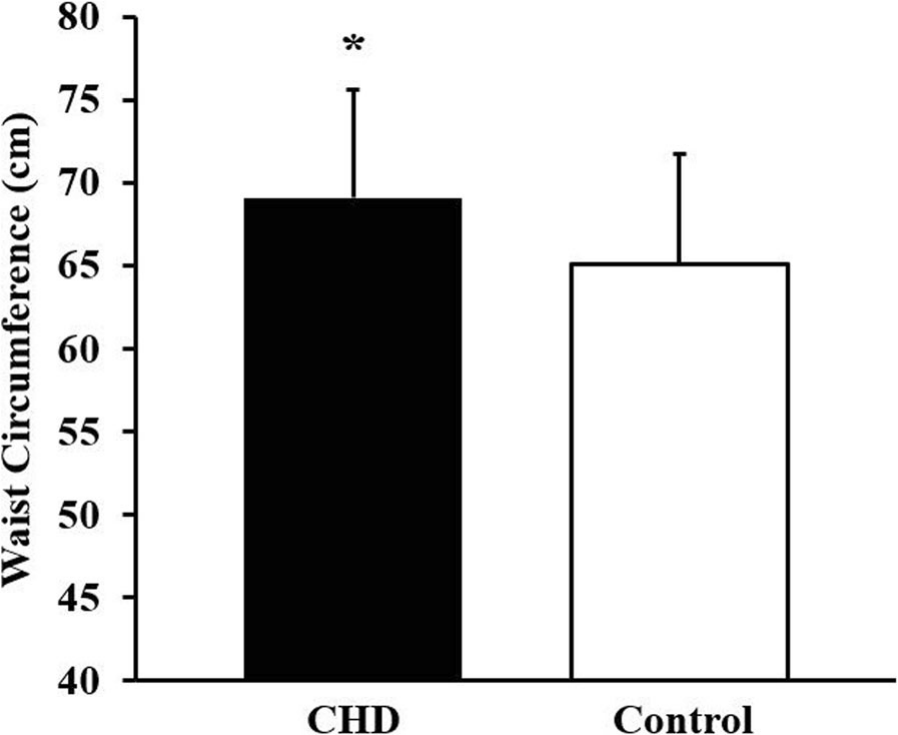
Adjusted mean and standard deviation of waist circumference in children/adolescents with CHD (congenital heart disease; *n* = 32) and controls (*n* = 23). Group differences assessed by ANCOVA with sex, birthweight, physical activity, and lean mass as co-variates. *Indicates *p* = 0.039

The analysis of covariance revealed that CHD participants had a significantly greater waist circumference than controls (estimated marginal mean difference = 3.9 cm, 69.1 ± 6.6 cm vs. 65.1 ± 6.5, respectively) when controlling for sex, birthweight, physical activity score, and total lean mass (*F* (1, 49) = 4.488, *p* = 0.039; Fig. 1).

## Discussion

The major novel finding of this study was that waist circumference was greater in children with diverse CHD lesions in comparison to healthy peers, independent of sex, birthweight, total lean mass, and physical activity. In addition, higher physical activity levels are related to lower waist circumference in children with CHD; however, the analysis of covariance finding indicates that high physical activity in children with CHD may not equalize waist circumference to that of healthy controls. While our smaller sample size warrants validation of findings in a larger cohort, the hypothesis generated from this study is potentially of great clinical importance. Elevated waist circumference and abdominal fat may drive or be one of the contributing mechanisms of cardiovascular and metabolic disease risk in this population.

### Importance of waist circumference determination in children with CHD

Studies have demonstrated that adipose tissue, particularly abdominal fat and specifically visceral fat, functions as an endocrine organ. Adipocytes, especially in those with metabolically-unhealthy obesity, secrete inflammatory cytokines, which may cause or exacerbate endothelial dysfunction and insulin resistance [37]. Waist circumference confers cardiovascular and metabolic disease risks above and beyond body fat or BMI, even in children with obesity [38, 39]. The implication of waist circumference on the health of individuals with CHD is suggested by findings in one of the few studies of cardiometabolic health in children with CHD by Zaqout et al. [40]. These investigators reported higher waist circumference, in addition to fasting insulin, and insulin resistance-homeostasis model in children with CHD compared to healthy controls, despite similar levels of objectively measured physical activity [40]. In one of the few studies specifically investigating waist circumference in children with CHD, Haapala et al. [26] measured children with chronic disease (including CHD) and found that waist circumference explained some of the relationship between arterial stiffness and decreased exercise capacity which was unaffected by sport participation (a surrogate measure of physical activity). These reports suggest that waist circumference may be a contributor, underpinning decreased fitness and elevated cardiovascular disease risk in pediatric clinical populations [26, 40]. Our study extends the prior work of Haapala et al. [41] by demonstrating that children with CHD have elevated waist circumference despite no difference in level of physical activity compared to healthy controls.

### Physical activity in children with CHD

Our findings indicate that physical activity scores were not significantly different between children with CHD and healthy controls. Consistent with our observations, a recent study by Stone et al. [42] in a younger population (3–5 years) with CHD reported similar levels of physical activity to healthy controls. The study by Zaqout et al. [40] showed greater self-reported physical activity levels in children with CHD compared to healthy controls but no difference in accelerometer-measured physical activity.

Our findings do; however, contrast those of Ray et al. [13] who reported that physical activity in children with CHD 10–14 years was lower than levels reported in age-matched healthy children. Discrepant findings may reflect the known error susceptibility of self-reporting physical activity, or differences between self-reported and objectively measured physical activity as in the study by Zaqout et al. [40]. Such discrepancies may also reflect recent changes to exercise prescription for children with CHD [12]. Indeed, previous recommendations that children with CHD should not participate in sport or leisure time physical activity to the same degree as healthy children has been replaced by encouraging 60 min of moderate-to-vigorous physical activity per day for optimal development [43].

### Effect of physical activity on waist circumference

The relationships between physical activity, adiposity, and waist circumference are well established in healthy adults, adolescents, and children [33, 44]. A prospective study in healthy Canadian children and adolescents by Carson et al. [45] found that waist circumference at baseline and at the end of the 2-year study was lower in the highest vigorous physical activity quartile after adjustment for age, sex, diet, accelerometer wear time, and time spent in other intensities of physical activity. Ekelund et al. [32] found a mean waist circumference difference of 2 cm between healthy children in the top and healthy children in the bottom tertiles of time spent in moderate-to-vigorous physical activity. We found a similar relationship but at a much greater magnitude with a 26 cm difference between the top and bottom quartile of physical activity with CHD and control groups combined. The difference between studies is likely due in part to the large difference in sample size as the study by Ekelund et al. [32] included data from over 20,000 children. In the current study, CHD was overrepresented in the lowest physical activity quartile potentially contributing to the large spread in the waist circumference values between tertiles; although, the difference in the distribution of CHD and controls over quartiles was not significant (data not shown). Our findings also indicate that the relationship between physical activity and waist circumference is stronger in controls than in CHD. We also identified a 3.9 cm difference in waist circumference between CHD and controls independent of physical activity. Taken together, these findings suggest that while physical activity influences waist circumference and contributes to the identified group difference in waist circumference, there are other factors involved. We suspect that waist circumference may be intrinsically linked (whether causally or associatively) with CHD and may contribute to population specific health disparities independent of level of physical activity.

### A mechanistic link between waist circumference and cardiovascular disease risk in CHD

One way in which waist circumference may be uniquely related to CHD is through early exposure to elevated psychological stress leading to inflammation. CHD is associated with signs of stress in infants and subjective feelings of stress in older children [46, 47]. Exposure to early life stress can permanently alter metabolic and adipose properties resulting in higher cortisol, and abdominal fat deposition in later life as demonstrated with rodent models [48]. Chronic social and cognitive stress is associated with elevated inflammatory markers such as interleukins and C-reactive protein. Mediated by the hypothalamo-pituitary-adrenal axis, this inflammatory processes is intimately linked with overweight, obesity, and abdominal fat and its sequelae of comorbidities including risk factors for cardiovascular disease and coronary artery disease [49]. Inflammation may play an early role in altered vascular dynamics as inflammatory cytokines are elevated in young children with CHD [50]. For example, in young children with CHD (up to 3 years), circulating inflammatory cytokines (macrophage migration inhibitory factor chemokine, T-cells, interleukin-17 and Th2 immune response mediator) are positively correlated with the severity of the disease, and with pulmonary vascular resistance and congestion [50]. Physical activity is known to decrease adiposity and BMI, and markers of stress and chronic inflammation, and to mitigate the association between stress and adiposity in otherwise healthy children [51–54]. Physical activity has similar health benefits in children with CHD [16, 40, 55–57]; thus early stress experienced by children with CHD may underlie findings of elevated waist circumference, and weaker associations between physical activity and waist circumference. The theory is compelling that there exists a cascade whereby early physical and psychological stress leads to elevated inflammation and cortisol, which drives elevated abdominal fat accrual, further contributing to inflammation and vascular alteration, which cannot be fully offset by physical activity, and ultimately leading to increased cardiovascular disease risk.

### Limitations

This study was a cross-sectional design with data from a small cohort of children and as such was unable to look at change over time or infer causation of elevated waist circumference in children with CHD. Our novel analysis was performed on our CHAMPS cohort and thus our sample size was driven based on participants with the needed metrics for the primary purposes of exploring possible waist circumference differences in the CHD study group. Given our observation of differences in waist circumference in our relatively small study, we are encouraged to explore further these observations in a larger and more thoroughly characterized CHD patient group. Dietary data was not available on participants. Elevated caloric intake or suboptimal food choices may be present in the CHD cohort due to a history of encouraging weight gain and thus may influence abdominal fat accrual. Furthermore, dietary supplements and patterns leading to hyperlipidemia can accelerate or mitigate the development of atherosclerosis and cardiovascular disease [58]. Waist circumference is a surrogate measure of abdominal fat, and physical activity was self-reported. Future studies should attempt to use objective measures for each. Although early stress experiences are likely in this cohort, measures of stress and their relationship to inflammation were not measured in this study, but should be pursued in the future. Finally, this was a heterogeneous group of children regarding the type and severity of CHD, and due to the small sample size, we could not control for lesion differences. Findings may not be generalizable to all CHD presentations.

### Clinical relevance: future cardiovascular disease risk for children with CHD

Abdominal fat accrual has strong associations with arterial stiffness and cardiovascular disease risk, and predicts left ventricular mass and diastolic function independent of BMI in healthy children and adults [21, 59–61]. Increased arterial stiffness [16] and abnormal cardiac function are common in children with various CHD lesions [62], and these disparities compared to healthy controls may worsen with age [63]. As such, children with CHD appear to be at an elevated risk of early-onset chronic cardiovascular diseases like hypertension and coronary artery disease, and metabolic diseases like type 2 diabetes [1]. With improvements in the long-term prognosis of children with CHD, the prevalence of these diseases in adult survivors is increasing [1, 40]. This increased life expectancy justifies the urgency of identifying the mechanisms behind disease disparities and factors to which these risks are amenable, such as lifestyle (e.g.*,* physical activity) and body composition. Abdominal adiposity (potentially driven by stress exposure) may be a key factor in disease development and progression in this population, thus studies exploring the role of waist circumference in the etiology of cardiovascular disease in the CHD population is clearly warranted.

## Conclusions

The results from the current study highlight the complexity of the relationship between waist circumference and physical activity. The potential role of waist circumference on the discrepancy in exercise capacity and tolerance, and arterial stiffness in this population needs further exploration, but it is supported by findings in healthy children in which the relationship between fitness and arterial stiffness is attenuated after adjustment for adiposity including waist circumference [7]. Indeed, physical activity and fitness may not ensure cardiovascular health if adiposity levels, particularly abdominal adiposity levels, are elevated. Further studies on waist circumference (and abdominal adiposity) in CHD with the ability to clarify this connection are needed in an attempt to aid in the prevention, detection, and appropriate treatment of cardiovascular disease in the CHD population [2].

## Data Availability

The datasets used and/or analyzed during the current study are available from the corresponding author on reasonable request.

## Abbreviations

BMI: Body mass index
CHAMPS: Children’s Healthy-Heart Activity Monitoring Program in Saskatchewan
CHD: Congenital heart disease
DXA: Dual-energy x-ray absorptiometry
